# Supplementary material for: Association between metabolically healthy obesity/overweight and cardiovascular disease risk: A representative cohort study in Taiwan
Source: PLoS One. 2021 Feb 1;16(2):e0246378. doi: 10.1371/journal.pone.0246378 (PMC7850496; doi:10.1371/journal.pone.0246378)
Supplement: S1 Table — (DOCX) [file pone.0246378.s001.docx]

**S1 Table. Definitions of hypertension, type 2 diabetes, hyperlipidemia in the study cohort.**

|  | Definition |
| --- | --- |
| Hypertension | (Systolic blood pressure ≥140 mmHg and diastolic blood pressure ≥90 mmHg) OR (prescription of the anti-hypertensive agents according to the ATC codes ≥28 tablets one year before the index date) |
| Type 2 diabetes | (Fasting glucose ≥126 mg/dL and HbA1c ≥6.5% in the 1^st^ survey ) OR (prescription of the anti-diabetic agents according to the ATC codes ≥28 tablets one year before the index date) |
| Hyperlipidemia | (Low density lipoprotein-cholesterol ≥160 mg/dL in the 1^st^ survey ) OR (prescription of the lipid-lowering agents according to the ATC codes ≥28 tablets one year before the index date) |

ATC code: anatomical therapeutic chemical code
