# Supplementary material for: Association between metabolically healthy obesity/overweight and cardiovascular disease risk: A representative cohort study in Taiwan
Source: PLoS One. 2021 Feb 1;16(2):e0246378. doi: 10.1371/journal.pone.0246378 (PMC7850496; doi:10.1371/journal.pone.0246378)
Supplement: S2 Table — (DOCX) [file pone.0246378.s002.docx]

**S2 Table. The anatomical therapeutic chemical codes used to define the medications in the study cohort.**

| Medication | Anatomical therapeutic chemical codes |
| --- | --- |
| Anti-hypertensive agents | Beta blocker, α2-agonist, α-blocker, sodium nitroprusside, diuretics , selective aldosterone receptor antagonist, calcium channel blocker, angiotensin-converting enzyme inhibitors, angiotensin receptor blocker, fixed-dose combinations, others (Minoxidil)  AC36675100,BC16080100,BC20238100,AB43852100,AC46867100,BC21303100,A031032100,A036844100,AB41956100,AC29301100,AC29313100,BC16888100,BC20174100,BC23799100,A041679100,AB29478100,AB294781G0,AB32322100,AB323221G0,AB33029100,AB330291G0,AB34359100,AB343591G0,AB361291G0,AB36130100,AB361301G0,AB38867100,AB388671G0,AB44843100,AB448431G0,AB515971G0,AC27452100,AC274521G0,AC29443100,AC294431G0,AC29735100,AC297351G0,AC31223100,AC312231G0,AC33124100,AC331241G0,AC35367100,AC353671G0,AC3612910,AC37928100,AC379281G0,AC39816100,AC398161G0,AC39843100,AC398431G0,AC41497100,AC414971G0,AC43598100,AC435981G0,AC43704100,AC437041G0,AC44163100,BC22144100,BC22158100,BC221591G0,BC221601G0,A042909100,AB41669100,AB47472100,AC42967100,AC42999100,BC06049100,AB44619100,AC45354100,BC21530100,BC22553100,AB45348100,AB47538100,AB48439100,AB55406100,AB55984100,AC48905100,AC49083100,AC49504100,AC49544100,AC49623100,AC4965010,AC49851100,AC49915100,AC50061100,BC17090100,BC17125100,BC24039100,BC26700100,A046026100,A046140100,AC30712100,AC31226100,AC32350221,AC39703100,AC49266100,AC49550221,BC17560100,BC17586100,BC24532100,AC44731100,AC46404100,AC46699100,AC47239100,AC47295100,AC47434100,AC47460100,AC47510100,AC47894100,AC48168100,AC48309100,AC49358100,AC49469100,AC55987100,AC57128100,AC57370100,BC20452100,BC22071100,BC24967100,BC25070100,BC25674100,AC29804100,AC34300100,A019039100,A020613100,A0214801G0,A0384871G0,AC14079100,AC14981209,AC21480100,AC21684100,AC23764100,AC26144100,AC38487100,BC25391100,AA49920100,AA57838100,AB44621100,AC26741100,AC267411G0,AC30415100,AC39962100,AC42378100,AC423781G0,AC42874100,AC43467100,AC43765100,AC44567100,AC44853100,AC448531G0,AC45013100,AC45039100,AC450391G0,AC45144100,AC50263100,AC57288100,AC57929100,AC59902100,AC599021G0,BC216411G0,BC216431G0,BC23179100,BC23711100,A005266100,A012898100,A025245209,A025245277,AC00178100,AC001781G0,AC02756100,AC07342100,AC18232100,AC30742100,AC30743100,AC30879100,AC31534100,AC315341G0,AC31540100,AC315401G0,AC32117100,AC321171G0,AC32851100,BC07538100,BC21879248,A016964100,A030456100,A031179100,AB03388100,AB033881G0,AC03098100,AC030981G0,AC04276100,AC042761G0,AC10917100,AC109171G0,AC14346100,AC40294100,AC46028100,AC460281G0,AC58612100,AC586121G0,N003316100,NC00141100,NC001411G0,NC001411GA,NC02597100,NC02791100,NC027911G0,NC05874100,NC08138100,NC081381G0,NC11948100,NC16648100,NC166481G0,A026027100,A027818100,A028200100,A046691100,A047171100,A047420100,A051388100,AB41641100,AB45955100,AB47372100,AC34090100,AC42380100,AC423801G0,AC42659100,AC42749100,AC43728100,AC44828100,AC46538100,AC473721G0,AC47606100,AC47789100,AC47996100,AC48122100,AC48705100,AC487051G0,AC50136100,AC50254100,AC50772100,BC16528100,BC21754100,BC223871G0,A0342831G0,AB27928212,AB30749100,AB307491G0,AB44046157,AB45363100,AB453631G0,AC01047100,AC010471G0,AC02368100,AC023681G0,AC11150212,AC22641100,AC226411G0,AC25164219,AC26154100,AC27244100,AC27577212,AC28079100,AC30521212,AC30697100,AC306971G0,AC14015100,AC18569100,AC185691G0,AC22908100,AC229081G0,AC25965100,AC259651G0,AC30526100,AC305261G0,AC41545100,AC415451G0,AC50116100,AC501161G0,AC501161G4,AC501161G5,AC501161G6,AC59370100,BC22610100,BC226101G0,BC24306100,BC27122100,BC27255100,A049307100,AA42704100,AA45670100,AB44098100,AB44468100,AB44475100,AB44607100,AB45392100,AB46480100,AB49969100,AB58075100,AB58278100,AC24018100,AC27439100,AC28131100,AC29436100,AC294361G0,AC32801100,AC328011G0,AC34553100,AC35891100,AC38154100,AC38996100,AC40168100,AC401681G0,AC41058100,AC42840100,AC42857100,AC42917100,AC43931100,AC43970100,AC44371100,AC44575100,AC44631100,AC44687100,AC44882100,AC44925100,AC45085100,AC45177100,AC45187100,AC45448100,AC45510100,AC46046100,AC47093100,AC47536100,AC47626248,AC47632100,AC48064100,AC48313100,AC48465100,AC48559100,AC48600100,AC48803212,AC48803229,AC48803240,AC48833100,AC48859100,AC48882229,AC48994100,AC49057100,AC49376100,AC49527100,AC49876100,AC49963100,AC49966100,AC50138100,AC52520100,AC52616100,AC55540100,AC55552100,AC55896100,AC56633100,AC56681100,AC56716100,AC56780100,AC57114100,AC57219100,AC57368100,AC58208100,AC58310100,AC58993229,AC58993238,AC59056100,AC59415100,AC59781263,AC60161212,BA21344100,BA22951100,BB24898100,BC17469100,BC17488100,BC18842100,BC18843248,BC19856100,BC198561G0,BC19884100,BC20021229,BC20254100,BC21149100,BC21571100,BC225931G0,BC23293100,BC24364100,BC25198100,BC25199100,BC26222229,BC26274100,BC26415100,BC27093100,BC27139100,A0398791G0,AC27307212,AC27775100,AC277751G0,AC28032100,AC30120100,AC301201G0,AC30615100,AC306151G0,AC31537100,AC315371G0,AC33822100,AC37229100,AC38926100,AC39879100,AC43071100,BC16350212,BC17118100,BC17292100,BC19953100,BC23708100,BC237081G0,AB31889100,AB38600100,AC10358100,AC103581G0,AC30629100,AC306291G0AC32876100,AC33224100,AC332241G0,AC34373100,AC35996100,AC359961G0,AC36966100,AC369661G0,AC38422100,AC45926100,AC46439100AC48127100,AC48128100,AC57887100,BC18540248,BC19885100,BC22678100,BC22997100,BC23001100,A036790100,A040779100,A040949100,A041281100,A042346100,A042711100,A042712100,A044315100,A044810100,AB36788100,AB367881G0,AB48836100,AB51029100,AB54966100,AC30452100,AC304521G0,AC32275100,AC322751G0,AC32706100,AC32711100,AC33910100,AC33915100,AC34598100,AC345981G0,AC34880100,AC348801G0,AC35420100,AC354201G0,AC35836100,AC37204100,AC37833100,AC37835100,AC38204100,AC382041G0,AC39401100,AC394011G0,AC39414100,AC394141G0,AC39974100,AC399741G0,AC41430100,AC42584100,AC425841G0,AC42824100,AC43210100,AC43330100,AC43950100,AC43951100,AC44161100,AC443151G0,AC44420100,AC444201G0,AC444201G2,AC44477100,AC44480100,AC444801G0,AC448101G0,AC45172100,AC45254100,AC452541G0,AC45975100,AC46199100,AC46995100,AC47002100,AC48119100,AC481191G0,AC48972100,AC49178100,AC49755100,AC49904100,AC50177100,AC54859100,AC55957100,AC57235100,AC57246100,AC57413100,AC58258100,AC59022100,BB25098100,BC19238100,BC19631100,BC19632100,BC22124100,BC22151100,BC22152100,BC23518100,BC23603100,BC24304100,BC24725100,BC24855100,A055915100,AA56318100,AA56320100,AA58268100,AB46315100,AB46661100,AB50240100,AB55028100,AB55557100,AB55585100,AB55931100,AB56670100,AB56697100,AB57103100,AB57178100,AB57204100,AB57234100,AB57343100,AB57415100,AB57864100,AC45203100,AC45847100,AC47911100,AC49610100,AC49739100,AC49835100,AC49887100,AC52436100,AC52593100,AC52614100,AC55296100,AC55531100,AC55558100,AC55950100,AC56689100,AC56745100,AC57232100,AC57318100,AC57342100,AC57380100,AC57381100,AC57410100,AC57821100,AC57909100,AC58085100,AC58090100,AC58169100,AC58170100,AC58190100,AC58191100,AC58199100,AC58231100,AC58235100,AC58273100,AC58537100,AC58596100,AC58641100,AC58768100,AC58834100,AC58841100,AC59032100,AC59196100,AC59197100,AC59199100,AC59214100,AC59260100,AC59278100,AC59407100,AC59736100,BA24634100,BA25589100,BB26409100,BB26577100,BC21914100,BC22551100,BC22843100,BC23128100,BC23161100,BC23162100,BC23373100,BC23374100,BC23655100,BC24497100,BC24645100,BC24655100,BC25005100,BC25095100,BC25197100,BC25210100,BC25342100,BC25756100,BC25766100,BC25879100,BC25897100,BC25965100,BC26321100,BC26322100,BC26369100,BC26391100,BC26446100,BC26463100,BC26464100,BC26472100,BC26503100,BC26557100,BC26659100,BC26820100,BC26830100,BC26967100,BC26992100,BC27253100,BC27433100,BC27440100,BC27441100,AC58370100,BC24823100,BC26467100,BC26468100,BC25492100,BC25493100,BC25494100,BC25495100,BC25496100,BC26670100,BC26671100,BC26672100,A056719100,AA48757100,AB50431100,AB57116100,AB57237100,AC48990100,AC52568100,AC57153100,AC57371100,AC58064100,AC59742100,AC59809100,AC59841100,BC22989100,BC23109100,BC23220100,BC23266100,BC23267100,BC23649100,BC23654100,BC23922100,BC24521100,BC24592100,BC24643100,BC24646100,BC24968100,BC24969100,BC25207100,BC25208100,BC25501100,BC26048100,BC26209100,BC26210100,BC26398100,BC26399100,BC26400100,BC26496100,BC26537100,BC27014100 |
| Anti-diabetic agents | Insulin, biguanide, sulfonylurea, α-glucosidase inhibitor, thiazolidinedione, glucagon-like peptide 1, sodium glucose co-transporters 2-inhibitor, dipeptidyl peptidase-4 inhibitors, fixed-dose combinations, others (Guar gum, Repaglinide, Nateglinide, Mitiglinide, calcium hydrate)  K000663299,K000739299,KC00595266,KC00596266,KC00663209,KC00729266,KC00739209,KC00795266,KC00803266,KC00823266,KC00898266,KC00899266,KC00900266,K000657299,K000760299,KC00657209,KC00760209,K000653299,KC00653209,KC00820266,KC00908266,KC00728266,KC00745209,KC00810266,KC00986266,KC01011272,KC01054266,KC01080216,A035324100,A0353241G0,A036349100,A038498100,A0384981G0,A039546100,A042464100,A0424641G0,A046686100,A047089100,A0488681G0,A050119100,AB34096100,AB340961G0,AB35665100,AB356651G0,AB408351G0,AB41233100,AB412331G0,AB43706100,AB437061G0,AB47532100,AB475321G0,AB47991100,AB48130100,AB48229100,AB48484100,AB484841G0,AB49457100,AB494571G0,AB49599100,AB52485100,AB524851G0,AB55258100,AB552581G0,AB55298100,AB56661100,AB56756100,AB567561G0,AB57217100,AB572171G0,AB58280100,AB582801G0,AC10323100,AC36201100,AC362011G0,AC36204100,AC362041G0,AC363491G0,AC36820100,AC368201G0,AC38680100,AC386801G0,AC40047100,AC400471G0,AC40098100,AC40119100,AC401191G0,AC40781100,AC407811G0,AC407811G4,AC407811G7,AC40835100,AC417501G0,AC42916100,AC429161G0,AC44233100,AC47086100,AC470861G0,AC47594100,AC47687100,AC479911G0,AC481301G0,AC482291G0,AC48734100,AC48863100,AC488631G0,AC48868100,AC48910100,AC49531100,AC495991G0,AC49659100,AC49957100,AC50080100,AC52534100,AC525341G0,AC552981G0,AC55985100,AC559851G0,AC56652100,AC566611G0,AC57171100,AC571711G0,AC57172100,AC571721G0,AC57177100,AC571771G0,AC57244100,AC57265100,AC57979100,AC58072100,AC58257100,AC58523100,AC585231G0,AC58534100,AC585341G0,AC58564100,AC585641G0,AC58613100,AC586131G0,AC589741G0,AC59009100,AC59654100,AC596541G0,AC59686100,AC596861G0,BA25308100,BA253081G0,BC071521G0,BC182311G0,BC22662100,BC226621G0,BC22663100,BC241891G0,BC25182100,BC25635100,BC26637100,BC271571G0,BC274021G0,BC274151G0,A003792100,A006823100,A022634100,A029325100,A029824100,A030512100,A0345501G0,A035254100,A035414100,A0354141G0,A036917100,A038681100,A040641100,A0406411G0,A042395100,A043051100,A043520100,A043858100,AB30970100,AB34736100,AB347361G0,AB40583100,AB405831G0,AB42908100,AB429081G0,AB43265100,AB432651G0,AB46074100,AB46658100,AB46766100,AB47070100,AB470701G0,AB47172100,AB48089100,AB49225100,AB49466100,AB55560100,AB57845100,AC02863100,AC028631G0,AC04715100,AC047151G0,AC20713100,AC207131G0,AC216401G0,AC226341G0,AC28245100,AC282451G0,AC29337100,AC293371G0,AC29513100,AC295131G0,AC30305100,AC303051G0,AC30698100,AC306981G0,AC309701G0,AC32831100,AC34348100,AC343481G0,AC34550100,AC34621100,AC346211G0,AC34893100,AC35670100,AC35795100,AC357951G0,AC35806100,AC36240100,AC362401G0,AC36395100,AC363951G0,AC36431100,AC37527100,AC375271G0,AC38181100,AC38500100,AC39144100,AC39408100,AC394081G0,AC39734100,AC397341G0,AC40233100,AC402331G0,AC41563100,AC41597100,AC41805100,AC42888100,AC43177100,AC435201G0,AC44172100,AC441721G0,AC44419100,AC45703100,AC46070100,AC46423100,AC464231G0,AC46640100,AC46647100,AC47049100,AC47231100,AC472311G0,AC47258100,AC47485100,AC47790100,AC47871100,AC48068100,AC480891G0,AC48121100,AC48307100,AC483071G0,AC48510100,AC485101G0,AC48560100,AC48846100,AC49072100,AC49114100,AC491141G0,AC492251G0,AC494661G0,AC50091100,AC50181100,AC501811G0,AC50769100,AC507691G0,AC52113100,AC54857100,AC55269100,AC57117100,AC57168100,AC57921100,AC579211G0,AC57989100,AC58088100,AC58121100,AC59396100,AC59625100,BB25266100,BC12873100,BC128731G0,BC224421G0,BC22671100,BC235031G0,A047680100,AB47671100,AB47811100,AB47848100,AB47981100,AB47984100,AB51714100,AB57312100,AC45646100,AC46488100,AC47307100,AC47878100,AC48095100,AC48228100,AC48855100,AC48898100,AC49204100,AC49494100,AC49579100,AC49697100,AC49829100,AC50084100,AC57326100,AC58092100,AC58241100,AC58388100,AC58975100,AC58976100,AC58977100,BB20786100,BB20787100,A049615100,A052589100,AA48333100,AA49116100,AA49429100,AA49500100,AA49930100,AA57769100,AB49581100,AB55275100,AC48007100,AC48057100,AC48098100,AC48516100,AC48602100,AC48753100,AC49085100,AC49119100,AC49549100,AC49560100,AC49625100,AC50043100,AC50130100,AC50426100,AC55011100,AC56669100,AC56692100,AC57872100,AC57897100,AC58350100,AC58410100,BC23206100,BC23207100,A044073121,A049924121,AC47403121,A049144100,AA49061100,AB46620100,AB49532100,AB52308100,AB57225100,AB57327100,AC47441100,AC48660100,AC49995100,AC58068100,BC22641100,BC26213100,BC26269100,AC47129100,AC47266100,AC47631100,AC47750100,AC50173100,BC23244100,BC23245100,AA52337100,BC25004213,BC25004297,KC00914216,BC27048263,BC27049263,KC00978206,KC00979206,BC26950100,BC26475100,BC26476100,BC26405100,BC26406100,BC27458100,BC26298100,BC26299100,BC26300100,BC25537100,BC25220100,BC25221100,AC58620100,AC59308100,AC60186100,BC24668100,AC59782100,BC25306100,A046732100,AB58071100,AC46733100,AC57799100,AC57860100,AC57861100,AC58954100,AC59300100,AC59393100,AC59759100,BA24876100,BC24005100,BC24006100,BC24839100,BC25041100,BC25043100,BC25453100,BC25454100,BC25455100,BC25480100,BC25481100,BC25482100,BC25792100,BC25793100,BC25794100,BC26681100,BC26682100,BC26683100,BC26685100,BC26686100,BC27035100,BC27036100,BC27037100,BC27038100,BC27039100,BC27040100,BC27073100,BC27074100,BC27114100,BC27115100,BC27116100,BC27117100 |
| Lipid-lowering agents | Statins, fibrate, cholestyramine resin , Probucol , Ezetimibe , proprotein convertase subtilisin/kexin type 9 inhibitors , fixed-dose combinations, others (Cholexamin, niacin)  A055967100,AB47348100,AC44998100,AC46402100,AC47775100,AC47907100,AC47924100,AC47928100,AC48608100,AC48813100,AC48926100,AC49190100,AC49360100,AC49535100,AC49661100,AC49672100,AC49699100,AC49792100,AC49841100,AC49997100,AC52465100,AC52479100,AC56804100,AC56806100,AC57176100,AC58207100,BC23970100,BC24339100,BC24868100,BC25211100,A042389100,AC39307100,AC39403100,AC39601100,AC42539100,AC42558100,AC42627100,AC43573100,B025412100,A043887100,A046022100,AB46029100,AB48586100,AB48644100,AB48681100,AB49021100,AB49143100,AB49454100,AB49503100,AC47341100,AC48469100,AC48513100,AC48684100,AC51523100,AC52581100,AC57126100,AC57741100,B024297100,BC23596100,BC23597100,AC56629100,BC21198100,BC21199100,BC23556100,BC26147100,AA48879100,AA49226100,AA49288100,AA49543100,AA56739100,AA57774100,AA57930100,AA57950100,AB51732100,AB54967100,AB57772100,AB57967100,AB58049100,AC50086100,AC51598100,AC52301100,AC52530100,AC55268100,AC55272100,AC55583100,AC55895100,AC55952100,AC55956100,AC56319100,AC56682100,AC56791100,AC57133100,AC57267100,AC57805100,AC58041100,AC58211100,AC58366100,AC58401100,AC58579100,BA25200100,BA25201100,BA25337100,BC22886100,BC22889100,BC22890100,BC26028100,BC26350100,BC27256100,BC27339100,AA57802100,AA57843100,AA57880100,AA58282100,AB57194100,AB57940100,AC57130100,AC57803100,AC57809100,AC58067100,AC58098100,AC58270100,AC58291100,AC58315100,AC58316100,AC58384100,AC58396100,AC58411100,AC58605100,AC58621100,AC58622100,AC58813100,AC58822100,AC59240100,AC59265100,AC59266100,AC59649100,AC59652100,AC60114100,AC60197100,BA25797100,BA25798100,BA26332100,BA26504100,BC24129100,BC24131100,BC24597100,BC25796100,BC26226100,BC26367100,BC26368100,BC26497100,BC26505100,BC26543100,BC26544100,BC26900100,BC27044100,AA57372100,AA58648100,AC58078100,AC58525100,AC58526100,AC58633100,AC58639100,AC59192100,AC59193100,AC59398100,AC60174100,AC60175100,BC25350100,BC27002100,A006865100,A027676100,A030590100,A036374100,A038102100,A042585100,AB31138100,AB38016100,AB41332100,AB413321G0,AB47228100,AB49551100,AC23998100,AC276761G0,AC29811100,AC30766100,AC31284100,AC31609100,AC31641100,AC31807100,AC31954100,AC32830100,AC32833100,AC328331G0AC32985100,AC33476100,AC33733100,AC33934100,AC35439100,AC35838100,AC358381G0,AC36483100,AC364831G0,AC37685100,AC38296100,AC39553100,AC39731100,AC41496100,AC41837100,AC41931100,AC419311G0,AC42244100,AC42619100,AC42775100,AC42826100,AC42990100,AC44461100,AC45374100,AC49333100,AC49808100,AC50087100,AC55927100,AC56720100,AC56754100,AC57730100,AC57944100,AC59363100,BC07125100,BC16094100,BC22654100,BC23215100,BC23770100,BC25594100,A036423127,AC35424127,AC28958100,BC17140100,A027087100,AC27449100,B020276100,BC24058100,BC26552100,BC27311100,YC00018209,AC57216100,AC59251100,BC24250100,BC26169100,BC26643100,BC27283100,BC27534100,BC27535100 |
