# Supplementary material for: Association between metabolically healthy obesity/overweight and cardiovascular disease risk: A representative cohort study in Taiwan
Source: PLoS One. 2021 Feb 1;16(2):e0246378. doi: 10.1371/journal.pone.0246378 (PMC7850496; doi:10.1371/journal.pone.0246378)
Supplement: S3 Table — (DOCX) [file pone.0246378.s003.docx]

**S3 Table. Definitions of the clinical outcomes in the study cohort.**

| Outcome | Definitions | ICD-9 codes | ICD-10 codes | Procedure codes |
| --- | --- | --- | --- | --- |
| Coronary heart disease | Either discharge diagnosis of ICD-9 codes or the procedure code | 410 | I21.01, I21.02, I21.09, I21.11, I21.19, I22.0, I22.1 | Revascularization percutaneous coronary intervention (33076B, 33077B, 33078B)  Coronary artery bypass graft (68023B, 68024B, 68025B)  N26002, N26003 |
|  |  | 411 | I20.0, I24.0, I24.1, I24.8, I24.9 |  |
|  |  | 414.00 | I25.10, I25.750, I25.751, I25.758, I25.759, I25.760, I25.761, I25. 768, I25.769, I25. 811, I25.812 |  |
|  |  | 414.01 | I25.10, I25.110, I25.111, I25.118, I25.119, I25.750, I25.751, I25.758, I25.759, I25. 811 |  |
|  |  | 410.02 | I25.710, I25.711, I25.718, I25.719, I25.812 |  |
|  |  | 410.03 | I25.730, I25.731, I25.738, I25.739 |  |
|  |  | 410.04 | I25.720, I25.721, I25.728, I25.729 |  |
|  |  | 414.05 | I25.700, I25.701, I25.708, I25.709, I25.730, I25.731, I25.738, I25.739, I25.760, I25.761, I25.768, I25.769, I25.790, I25.791, I25.798, I25.799, I25.810, I25.812 |  |
|  |  | v45.81 | Z95.1 |  |
|  |  | v45.82 | Z95.5, Z95.8, Z98.61 |  |
| Ischemia stroke | Discharge diagnosis of the ICD-9 codes | 433 | 165.1,,I63.02, I63.12, I65.21, I63.22, I65.1, I65.23, I65.29 , I63.031, I63.032, I63.039, I63.131, I63.132, I63.139, I63.231, I63.232, I63.239, I65.01, I65.02, I65.03, , I65.09, I63.011, I63.012, I63.019, I65.22, I63.111, I63.112, I63.119, I63.211, I63.212, I63.219, I65.8, 163.09, I63.19, I63.59, I65.9, I63.00, 163.10, I63.20, I63.29 |  |
|  |  | 434 | I66.01, I66.02, I66.03, I66.09, I66.11, I66.12, I66.13, I66.19, I66.21, I66.22, I66.23, I66.29, I66.3, I63.30, I63.311, I63.312, I63.319, I63.321, I63.322, I63.329, I63.331, I63.332, I63.339, I63.341, I63.342, I63.349, I63.39, I63.6, I66.01, I66.02, I66.03, I66.09, I66.11, I66.12, I66.13, I66.19, I66.21, I66.22, I66.23, I66.29, I66.3, I66.9, I66.40, I66.411, I66.412, I66.419, I66.421, I66.422, I66.429, I66.431, I66.432, I66.439, I66.441, I66.442, I66.449, I66.49, I66.01, I66.02, I66.03, I66.09, I66.11, I66.12, I66.13, I66.19, I66.21, I66.22, I66.23, I66.29, I66.3, I66.8, I66.9, I63.50, I63.511, I63.512, I63.519, I63.521, I63.522, I63.529, I63.531, I63.532, I63.539, I63.541, I63.542, I63.549, I63.59, I63.8, I63.9 |  |
|  |  | 435 | G45.0,G45.8,G45.1,G45.2, G46.0, G46.1, G46.2, G45.9, I67.841, I67.848 |  |
|  |  | 436 | I67.89 |  |
|  |  | 437.1 | I67.81, I67.82, I67.89 |  |
|  |  | 437.9 | I67.9 |  |

ICD-9: International Classification of Diseases-9^th^ revision; ICD-10: International Classification of Diseases-10^th^ revision
