# Supplementary material for: Association between metabolically healthy obesity/overweight and cardiovascular disease risk: A representative cohort study in Taiwan
Source: PLoS One. 2021 Feb 1;16(2):e0246378. doi: 10.1371/journal.pone.0246378 (PMC7850496; doi:10.1371/journal.pone.0246378)
Supplement: S4 Table — (DOCX) [file pone.0246378.s004.docx]

**S4 Table. Definitions of the covariates in the study cohort.**

| Covariates | Question in the questionnaire | Category |
| --- | --- | --- |
| Sex |  | Women/Men |
| Age | (Years old) | ≥20 to <40, ≥40 to <65, ≥65 |
| Smoking status | Have you smoked before? 1. Never; 2. Yes, but only a few times; 3. Yes, but not more than 5 packs (100 cigarettes) in my entire life; 4. Yes, more than 5 packs (100 cigarettes) in my entire life. If the answer is 4, than ask: Did you smoking in the recent one month? 1. Almost every day; 2. Occasionally; 3. Quitted. 1, 2 = smoker | Smoker/Non-smoker |
| Alcohol use | Do you drink alcohol? | No/Yes |
| Regular exercise habit | Exercises including running, swimming, ball sports, stair climbing and brisk walking.  In the past, have you exercised at least 3 times a week for at least 30 minutes for a period of more than 3 months? | Non-regular/Regular |
| Parental history of cardiovascular disease | Has your father ever had a heart attack? Has your father ever had a stroke?  Has your mother ever had a heart attack? Has your mother ever had a stroke? | No/Yes |
| Marital status | Single: unmarried; divorced or separated: married, but not living with a spouse, divorced, (official) separated, widowed, not mentioned, others  Living with spouse: Married, and living with a spouse, cohabitation | Single, divorced, separated/  Living with spouse |
| Education levels | Years of educated | <9 years/ ≥9 years |
| Average month income | (in New Taiwan dollar) | <40,000/ ≥40,000 |
| Low-density lipid cholesterol | (mg/dL) | Continuous variable |
