# Supplementary material for: Association between metabolically healthy obesity/overweight and cardiovascular disease risk: A representative cohort study in Taiwan
Source: PLoS One. 2021 Feb 1;16(2):e0246378. doi: 10.1371/journal.pone.0246378 (PMC7850496; doi:10.1371/journal.pone.0246378)
Supplement: S5 Table — (DOCX) [file pone.0246378.s005.docx]

**S5 Table. Baseline characteristics of metabolically healthy obesity and metabolically healthy overweight participants.**

|  | Metabolically healthy (n =491) | | Metabolically healthy (n =1,720) | |  |
| --- | --- | --- | --- | --- | --- |
| Characteristics | Overweight  (n= 358) | Obesity  (n= 133) | Overweight  (n= 1,054) | Obesity  (n= 666) | *p* value |
|  | Mean (SD) | Mean (SD) | Mean (SD) | Mean (SD) |  |
| Age (years old) | 44.6 (13.1) | 40.1 (12.4) | 49.6 (14.1) | 47.5 (15.1) | **<0.001** |
| Body mass index (kg/m^2^ ) | 25.2 (0.8) | 29.6 (4.0) | 25.4 (0.9) | 29.7 (2.5) | **<0.001** |
| Waist circumference (cm) | 83.5 (6.7) | 92.5 (8.4) | 86.6 (6.9) | 95.7 (8.4) | **<0.001** |
| Systolic blood pressure (mmHg) | 109.4 (9.7) | 112.9 (8.9) | 124.1 (17.6) | 128.2 (17.6) | **<0.001** |
| Diastolic blood pressure (mmHg) | 72.5 (6.9) | 74.2 (6.4) | 80.7 (11.2) | 84.0 (11.3) | **<0.001** |
| Fasting plasma glucose (mg/dL) | 87.4 (6.4) | 88.1 (6.6) | 102.2 (35.1) | 107.4 (41.3) | **<0.001** |
| Hemoglobulin A1c (%) | 5.15 (0.52) | 5.22 (0.48) | 5.66 (1.24) | 5.86 (1.32) | **<0.001** |
| Total cholesterol (mg/dL) | 182.2 (26.2) | 184.8 (28.6) | 196.3 (40.6) | 200.8 (44.3) | **<0.001** |
| Triglycerides (mg/dL) | 94.1 (28.1) | 99.5 (26.9) | 178.0 (102.7) | 193.8 (109.8) | **<0.001** |
| High-density lipoprotein cholesterol (mg/dL) | 60.8 (11.5) | 60.8 (11.8) | 50.1 (15.7) | 48.8 (15.8) | **<0.001** |
| Low-density lipoprotein cholesterol (mg/dL) | 113.2 (20.0) | 114.9 (20.5) | 126 (28.4) | 129.3 (29.1) | **<0.001** |
|  | n (%) | n (%) | n (%) | n (%) |  |
| 20-39 (years old) | 134 (37.4) | 63 (47.0) | 273 (25.9) | 220 (33.1) | **<0.001** |
| 40-64 (years old) | 197 (54.9) | 68 (50.8) | 599 (56.9) | 345 (51.9) | **<0.001** |
| ≥ 65 (years old) | 28 (7.8) | 3 (2.3) | 181 (17.2) | 100 (15.0) | **<0.001** |
| Women | 194 (54.0) | 67 (50.0) | 637 (60.4) | 403 (60.5) | **<0.001** |
| Smokers | 63 (17.6) | 31 (23.1) | 284 (27.0) | 196 (29.5) | **<0.001** |
| Alcohol used | 110 (30.6) | 35 (26.1) | 348 (33.1) | 218 (32.8) | **<0.001** |
| Regular exercise habit^a^ | 101 (28.2) | 30 (22.4) | 277 (26.3) | 144 (21.7) | **<0.001** |
| Menopaused status | 56 (15.6) | 21 (15.7) | 227 (21.6) | 149 (22.4) | **0.008** |
| Parental history of CVD | 87 (24.2) | 21 (15.8) | 291 (27.6) | 159 (23.9) | **<0.001** |
| Living with spouse | 269 (74.9) | 86 (64.2) | 804 (76.4) | 471 (70.8) | **<0.001** |
| Educational level ( ≥ 9 years of schooling) | 203 (56.6) | 75 (56.0) | 466 (44.3) | 295 (44.4) | **<0.001** |
| Average month income ≥ 40,000 NTD | 110 (30.6) | 28 (20.9) | 246 (23.4) | 138 (20.8) | **<0.001** |

Normal weight, 18.5 to 23.9 kg/m^2^; obesity/overweight, ≥24.0 kg/m^2^

^a^Regular exercise defined as more than 30 minutes a day, three times a week, lasting at least three months

SD: standard deviation
