# Supplementary material for: Association between metabolically healthy obesity/overweight and cardiovascular disease risk: A representative cohort study in Taiwan
Source: PLoS One. 2021 Feb 1;16(2):e0246378. doi: 10.1371/journal.pone.0246378 (PMC7850496; doi:10.1371/journal.pone.0246378)
Supplement: S6 Table — (DOCX) [file pone.0246378.s006.docx]

**S6 Table. Subgroup analyses for fatal and nonfatal cardiovascular disease risk by metabolic statuses and body mass index categories by different reference groups.**

|  | Metabolically healthy | | Metabolically unhealthy | |
| --- | --- | --- | --- | --- |
| Variables | Normal weight | **Obesity/overweight** | Normal weight | Obesity/overweight |
| **Sex** |  |  |  |  |
| Women | 1 | **5.00 (1.74, 14.41)** | **5.77 (2.28, 14.59)** | **8.82 (3.54, 21.92)** |
| Men | **7.42 (2.81, 19.58)** | **8.96 (3.21, 25.07)** | **13.78 (5.51, 34.45)** | **15.15 (6.11, 37.54)** |
| **Age** |  |  |  |  |
| <65 years old | 1 | 2.06 (0.99, 4.28) | **4.47 (2.57, 7.78)** | **5.65 (3.31, 9.63)** |
| ≥65 years old | **16.04 (7.75, 33.22)** | **30.74 (14.42, 65.57)** | **21.63 (12.32, 37.97)** | **24.94 (14.30, 43.50)** |
