# Supplementary material for: Association between metabolically healthy obesity/overweight and cardiovascular disease risk: A representative cohort study in Taiwan
Source: PLoS One. 2021 Feb 1;16(2):e0246378. doi: 10.1371/journal.pone.0246378 (PMC7850496; doi:10.1371/journal.pone.0246378)
Supplement: S7 Table — (DOCX) [file pone.0246378.s007.docx]

**S7 Table. Sensitivity analyses of the risk of fatal and nonfatal cardiovascular disease according to the metabolic status and anthropometric categories.**

| Sensitivity analyses | Metabolically healthy | | Metabolically unhealthy | |
| --- | --- | --- | --- | --- |
|  | Normal weight | Obesity/overweight | Normal weight | Obesity/overweight |
| **Excluding events in the first year** | 1 | **1.74 ( 1.02, 2.99)** | **2.39( 1.59, 3.59)** | **2.94 ( 1.98, 4.37)** |
| **Using calibrated BMI^a^** | 1 | **1.73 ( 1.01, 2.96)** | **2.49 ( 1.66, 3.73)** | **3.06 ( 2.06, 4.54)** |
| **Redefined cardiometabolic diseases^b^** | 1 | **1.72 ( 1.01, 2.91**) | **2.56 ( 1.71, 3.84)** | **3.08 ( 2.08, 4.57)** |
| **Redefined cardiometabolic diseases^c^** | 1 | **1.70 ( 1.00, 2.88**) | **2.51 ( 1.67, 3.76)** | **3.04 ( 2.05, 4.51)** |

Normal weight, 18.5 to 23.9 kg/m^2^ , obesity/overweight, ≥ 24.0 kg/m ^2^ ; optimal waist circumference, <80cm in women and <90 cm in men; abdominal obesity, ≥80cm in women and ≥ 90 cm in men

Adjusted for by model 3, presented with hazard ratio with 95% confidence interval

^a^Calibrated BMI = 0.355 + 0.985 * self-reported BMI in men; -0.316 + 1.02* self-reported BMI in women

^b^(Laboratory data in the 1^st^ survey) AND (prescription)

|  | Redefined definition |
| --- | --- |
| Hypertension | (Systolic blood pressure ≥140 mmHg and diastolic blood pressure ≥90 mmHg) **AND** (prescription of the anti-hypertensive agents according to the ATC codes ≥28 tablets one year before the index date) |
| Type 2 diabetes | (Fasting glucose ≥126 mg/dL and HbA1c ≥6.5% in the 1^st^ survey ) **AND** (prescription of the anti-diabetic agents according to the ATC codes ≥28 tablets one year before the index date) |
| Hyperlipidemia | (Low density lipoprotein-cholesterol ≥160 mg/dL in the 1^st^ survey ) **AND** (prescription of the lipid-lowering agents according to the ATC codes ≥28 tablets one year before the index date) |

ATC code: anatomical therapeutic chemical code

^c^(Laboratory data in the 1^st^ survey) AND (ICD-9 codes)

|  | Redefined definition |
| --- | --- |
| Hypertension | (BP ≥140/90 mmHg) **AND (ICD-9 codes** of 401-405, 437.2 from more than twice clinic visits or any discharge note within one year) |
| Type 2 diabetes | (Fasting glucose ≥126 mg/dL and HbA1c ≥6.5% in the 1^st^ survey) **AND (ICD-9 codes** of 250 from more than twice clinic visits or any discharge note within one year) |
| Hyperlipidemia | (Low density lipoprotein-cholesterol ≥160 mg/dL in the 1^st^ survey) **AND (ICD-9 codes** of 272 from more than twice clinic visits or any discharge note within one year) |

ICD-9: International Classification of Diseases, 9^th^ revision
