# Supplementary material for: Association between metabolically healthy obesity/overweight and cardiovascular disease risk: A representative cohort study in Taiwan
Source: PLoS One. 2021 Feb 1;16(2):e0246378. doi: 10.1371/journal.pone.0246378 (PMC7850496; doi:10.1371/journal.pone.0246378)
Supplement: S8 Table — (DOCX) [file pone.0246378.s008.docx]

**S8 Table. Sensitivity analyses of the risk of fatal and nonfatal cardiovascular disease in metabolically healthy obesity and metabolically healthy overweight participants.**

|  | Metabolically healthy | |  | Metabolically unhealthy | |
| --- | --- | --- | --- | --- | --- |
| Variables | Normal weight | Overweight | Obesity | Normal weight | Obesity/overweight |
| Participants | 1,479 | 358 | 133 | 1,668 | 1,720 |
| Person-years | 19,786.7 | 4722.4 | 1799.6 | 20,715.6 | 20,841.6 |
| Events | 30 | 23 | 3 | 156 | 229 |
| Incidence rate  (per 1,000 person-years) | 1.52 | 4.87 | 1.67 | 7.53 | 10.97 |
| Model 1 | 1 | **2.00 (1.16, 3.48)** | 1.02 (0.31, 3.35) | **2.70 (1.82, 4.00)** | **3.50 (2.38, 5.14)** |
| Model 2 | 1 | **2.02 (1.16, 3.51)** | 0.99 (0.30, 3.26) | **2.64 (1.78, 3.92)** | **3.34 (2.27, 4.91)** |
| Model 3 | 1 | **2.03 (1.17, 3.53)** | 0.97 (0.30, 3.19) | **2.48 (1.65, 3.71)** | **3.09 (2.08, 4.58)** |
