# Supplementary material for: Association between metabolically healthy obesity/overweight and cardiovascular disease risk: A representative cohort study in Taiwan
Source: PLoS One. 2021 Feb 1;16(2):e0246378. doi: 10.1371/journal.pone.0246378 (PMC7850496; doi:10.1371/journal.pone.0246378)
Supplement: S1 Fig — (DOCX) [file pone.0246378.s009.docx]

**S1 Fig. The logarithm negative logarithm plot against logarithm of time for proportional hazard assumption**

**
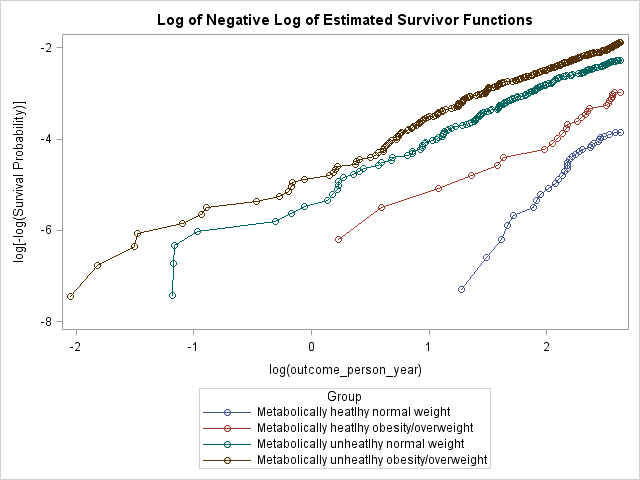
**
