# Supplementary material for: Association between metabolically healthy obesity/overweight and cardiovascular disease risk: A representative cohort study in Taiwan
Source: PLoS One. 2021 Feb 1;16(2):e0246378. doi: 10.1371/journal.pone.0246378 (PMC7850496; doi:10.1371/journal.pone.0246378)
Supplement: S2 Fig — (DOCX) [file pone.0246378.s010.docx]

**S2 Fig. The flow diagram of the participants enrollment.**

**
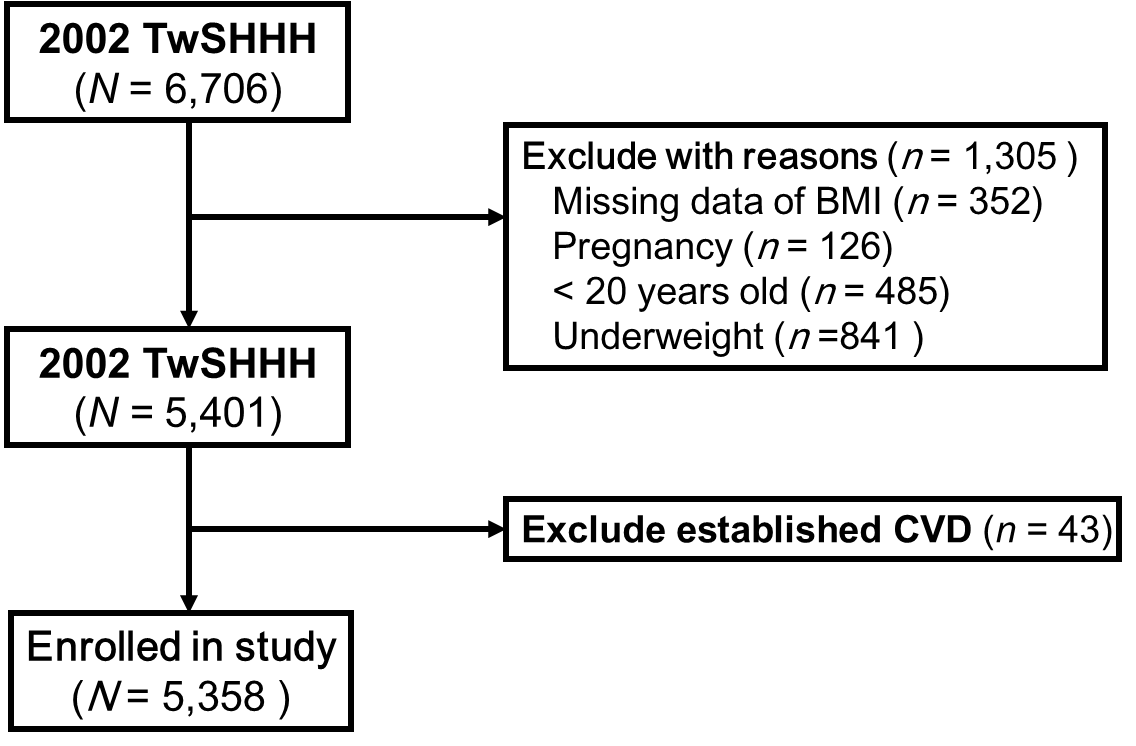
**

BMI: body mass index; CVD: cardiovascular disease; TwSHHH: Taiwanese Survey on Hypertension, Hyperglycemia, and Hyperlipidemia
