# Supplementary material for: Association between metabolically healthy obesity/overweight and cardiovascular disease risk: A representative cohort study in Taiwan
Source: PLoS One. 2021 Feb 1;16(2):e0246378. doi: 10.1371/journal.pone.0246378 (PMC7850496; doi:10.1371/journal.pone.0246378)
Supplement: S1 Raw data — (DOCX) [file pone.0246378.s011.docx]

The raw data was owned by the third party: Healthy and Welfare Data Science Center, HWDC

**SAS code**

libname data "E:\H108044\Yeh\data set";

libname w "E:\H108044\Yeh\data work";

libname w1 "E:\H108044\Yin\3 Data work";

/*raw data*/

**proc** **contents** data=h_bhp91.h_bhp_twhhhbas91 position; **run**; /*7578*/

**proc** **contents** data=h_bhp90.pa_id90 position; **run**; /*6706*/

**data** w.h_bhp_twhhh_v3;

set h_bhp_twhhh_v4;

/*outcome1*/

if Stroke_1=**1** or Stroke_death=**1** or cad_1=**1** or cad_death=**1** then outcome1=**1**; else outcome1=**0**;

if (Stroke_1=**1** and Stroke_day<=index_day) or (Stroke_death=**1** and death_day<=index_day) then outcome1=**.**;

if (cad_1=**1** and cad_day<=index_day) or (cad_death=**1** and death_day<=index_day) then outcome1=**.**;

if death=**1** and (death_day<Stroke_day or death_day<cad_day) then outcome1=**.**;

if outcome1=**1** then outcome1_py=(min(Stroke_day,cad_day,death_day)-index_day)/**365.25**;

if outcome1=**0** and death^=**1** then outcome1_py=(mdy(**12**,**31**,**2015**)-index_day)/**365.25**;

if outcome1=**0** and death=**1** then outcome1_py=(death_day-index_day)/**365.25**;

**data** h_bhp_twhhh_v33;

set w.h_bhp_twhhh_v3;

if BH in ("-999" "999.8") then BH=**.**;

if BW in ("-999" "999.8") then BW=**.**;

if BH=**.** then bh=height2007;

if BH in ("99998" "99999") then BH=**.**;

if pregnant in ("-9999" "-999") then pregnant=**.**;

BMI=BW/((BH/**100**)****2**);

if gender=**0** and pregnant=**1** then delete; /*n=126*/

if age<**20** then delete; /*n=485*/

/* if age<65 then delete; /*n=878*/

if bh=**.** or bw=**.** then delete; /*n=352*/

/* underweight n=841*/

If BMI <**18.5** then delete;

/* age*/

If **20**<=age<**40** then agec=**0**;

else if **40**<=age<**65** then agec=**1**;

else if age>=**65** then agec=**2**;

if agec in ("0" "1") then agec65=**0**; else agec65=**1**; /*subgroup*/

If agec65=**1** and **65**<=age<**75** then agec14=**0**; else agec14=**1**; /*subgroup*/

/*smoke*/ if (smoke= **4**) and (current_smoke in (**1**,**2**)) then smoker=**1**; else smoker =**0**;

/* Alcohol--alc */

/*exercise*/ if PA=**1** then exercise=**1**; else exercise=**0**;

/* menopause*/ if gender=**0** and menopause =**1** then menopausec=**1**; else menopausec=**0**;

/* HRT*/ if gender=**0** and HRT in ("2" "3") then HRTuse=**1**; else HRTuse=**0**;

/* CVDFHx*/

**%macro** aa(b);

&b._1=&b.***1**;

if &b. in ("2" "9") then &b._1=**0**;

**%mend** aa;

%***aa***(FatherCAD);

%***aa***(Fatherstroke);

%***aa***(MotherCAD);

%***aa***(Motherstroke);

if FatherCAD_1=**1** or Fatherstroke_1=**1** or MotherCAD_1 =**1** or Motherstroke_1=**1** then CVDFHx=**1**; else CVDFHx=**0**;

/*education*/

if **10**<=education<=**16** or **91**<=education<=**92** then edu=**1**;

else edu=**0**;

if education in ("-999" "96") then edu=**.**;

/*marriage*/

if marriage in (**1**,**6**) then marry=**1**; /*Living with spouse*/

else marry =**0**; /*single or divorced or seperated*/

/*income*/

if **1**<=income<=**6** then incomec=**0**; /*<40000*/

else incomec=**1**; /*>=40000*/

if income in ("-999" "95" "96" "98") then incomec=**0**;

/* BMI 3groups*/

If **0**<=BMI <**18.5** then BMIc=**1**;

Else If **18.5**<=BMI<**24** then BMIc=**2**;

Else If **24**<=BMI then BMIc=**3**;

/* BMI 4 groups*/

If **0**<=BMI <**18.5** then BMIc1=**1**;

Else If **18.5**<=BMI<**24** then BMIc1=**2**;

Else If **24**<=BMI<**27** then BMIc1=**3**;

Else If **27**<=BMI then BMIc1=**4**;

/*calibrated BMI*/

if gender=**1** then calBMI1=**0.355** + **0.985** * BMI;

else if gender=**0** then calBMI1=-**0.316** + **1.02***BMI ;

if gender=**1** then calBMI2=**0.17**+**0.986***BMI+**0.004***age;

else if gender=**0** then calBMI2=-**0.552**+ **1.011*** BMI +**0.01***age;

If **0**<=calBMI1 <**18.5** then cal1BMIc=**1**;

else If **18.5**<=calBMI1<**24** then cal1BMIc=**2**;

else If **24**<=calBMI1 then cal1BMIc=**3**;

If **0**<=calBMI2 <**18.5** then cal2BMIc=**1**;

else If **18.5**<=calBMI2<**24** then cal2BMIc=**2**;

else If **24**<=calBMI2 then cal2BMIc=**3**;

/* Waist circumference*/

if **0**<wc_value<**40** then wc_value=wc_value***2.54**;

if WC_value in ("999.9") then WC_value=**.**;

if (gender=**0** AND WC_value>=**80**) or (gender=**1** and WC_value>=**90**) then WC=**1**; else WC=**0**;

/*lab*/

if n_Glu in ("999" "99.9" "999.9") then n_Glu=" ";

if N_HB in ("999" "99.9" "999.9") then N_HB=" ";

if N_CHOL in ("999" "99.9" "999.9") then N_CHOL=" ";

if n_tri in ("999" "99.9" "999.9") then n_tri=" ";

if n_hdl in ("999" "99.9" "999.9") then n_hdl=" ";

if n_LDL in ("999" "99.9" "999.9") then n_LDL=" ";

if N_CREAT in ("999" "99.9" "999.9") then N_CREAT=" ";

if sbp1 in ("999" "99.9" "999.9") then sbp1=**.**;

if sbp2 in ("999" "99.9" "999.9") then sbp2=**.**;

if dbp1 in ("999" "99.9" "999.9") then dbp1=**.**;

if dbp2 in ("999" "99.9" "999.9") then dbp2=**.**;

Glu=n_Glu***1**;

HbA1c=N_HB***1**;

TC=N_CHOL***1**;

TG= n_tri***1**;

HDL=n_hdl***1**;

LDL=n_LDL***1**;

Cr=N_CREAT***1**;

/* sbp and dbp */

If sbp1 ne **.** and sbp2 ne **.** then sbp=(sbp1+sbp2)/**2**;

Else if sbp1=**.** Then sbp=sbp2;

Else if sbp2=**.** Then sbp=sbp1;

If dbp1 ne **.** and dbp2 ne **.** then dbp=(dbp1+dbp2)/**2**;

Else if dbp1=**.** Then dbp=dbp2;

Else if dbp2=**.** Then dbp=dbp1;

if (Glu>=**126** AND HbA1c>= **6.5**) or (DM_drug=**1**) then DM=**1**; else DM=**0**;

if (LDL>=**160**) or (LIPID_drug=**1**) then HL=**1**; else HL=**0**;

if (SBP>=**140**) AND (DBP>=**90**) or HTN_drug=**1** then HTN=**1**; else HTN=**0**;

If (DM=**1** or HL=**1** or HTN=**1**) then CM=**1**; else CM=**0**;

/* MH lab*/

if **0**<sbp<**130** and **0**<dbp<**85** then MHBP=**1**; else MHBP=**0**;

if **0**<Glu<**100** then MHGlu=**1**; else MHGlu=**0**;

if **0**<TG<**150** then MHTG=**1**; else MHTG=**0**;

if (gender=**1** and hdl>=**40**) or (gender=**0** and hdl >=**50**) then MHHDL=**1**; else MHHDL=**0**;

if MHBP=**1** AND MHGlu=**1** AND MHTG=**1** AND MHHDL=**1** then MHlab=**1**; else MHlab=**0**;

if (CM= **0**) AND MHlab=**1** then MH=**1**; else MH=**0**;

/*bmi */

if MH=**1** and BMIc=**2** then MHMU4=**0**;

else if MH=**1** and BMIc=**3** then MHMU4=**1**;

else if MH=**0** and BMIc=**2** then MHMU4=**2**;

else if MH=**0** and BMIc=**3** then MHMU4=**3**;

/*wc 4*/

if MH=**1** and WC=**1** then MHWC=**1**;

else if MH=**1** and WC=**0** then MHWC=**0**;

else if MH=**0** and WC=**1** then MHWC=**2**;

else if MH=**0** and WC=**0** then MHWC=**3**;

/*calBMI1 /

if MH=**1** and cal1BMIc=**1** then MHMU_cal16=**1**;

else if MH=**1** and cal1BMIc=**2** then MHMU_cal16=**0**;

else if MH=**1** and cal1BMIc=**3** then MHMU_cal16=**2**;

else if MH=**0** and cal1BMIc=**1** then MHMU_cal16=**3**;

else if MH=**0** and cal1BMIc=**2** then MHMU_cal16=**4**;

else if MH=**0** and cal1BMIc=**3** then MHMU_cal16=**5**;

/*calBMI2 */

if MH=**1** and cal2BMIc=**1** then MHMU_cal26=**1**;

else if MH=**1** and cal2BMIc=**2** then MHMU_cal26=**0**;

else if MH=**1** and cal2BMIc=**3** then MHMU_cal26=**2**;

else if MH=**0** and cal2BMIc=**1** then MHMU_cal26=**3**;

else if MH=**0** and cal2BMIc=**2** then MHMU_cal26=**4**;

else if MH=**0** and cal2BMIc=**3** then MHMU_cal26=**5**;

/*Sensitivity analysis for re-defined CM disease

/* DM sensitivity*/

if (Glu>=**126** and HbA1c>= **6.5**) and (DM_drug=**1**) then DM1=**1**; else DM1=**0**;

if (Glu>=**126** and HbA1c>= **6.5**) and (DM_dis_1="1" and DM_dis_day<=index_day) then DM5=**1**; else DM5=**0**;

/* HL sensitivity*/

if (LDL>=**160**) and (LIPID_drug=**1**) then HL1=**1**; else HL1=**0**;

if (LDL>=**160**)and (hl_dis_1="1" and hl_dis_day<=index_day) then HL5=**1**; else HL5=**0**;

/* HTN sensitivity*/

if (SBP>=**140** and DBP>=**90**) and HTN_drug=**1** then HTN1=**1**; else HTN1=**0**;

if (SBP>=**140** and DBP>=**90**) and (htn_dis_1="1" and htn_dis_day<=index_day) then HTN5=**1**; else HTN5=**0**;

/* CM*/

If (DM=**1** or HL=**1** or HTN=**1**) then CM=**1**; else CM=**0**;

If (DM1=**1** or HL1=**1** or HTN1=**1**) then CM1=**1**; else CM1=**0**;

If (DM5=**1** or HL5=**1** or HTN5=**1**) then CM5=**1**; else CM5=**0**;

/*MH*/

if (CM= **0**) AND MHlab=**1** then MH=**1**; else MH=**0**;

if (CM1= **0**) AND MHlab=**1** then MH1=**1**; else MH1=**0**;

if (CM5= **0**) AND MHlab=**1** then MH5=**1**; else MH5=**0**;

if MH1=**1** and BMIc=**1** then MHMUs1=**1**;

else if MH1=**1** and BMIc=**2** then MHMUs1=**0**;

else if MH1=**1** and BMIc=**3** then MHMUs1=**2**;

else if MH1=**0** and BMIc=**1** then MHMUs1=**3**;

else if MH1=**0** and BMIc=**2** then MHMUs1=**4**;

else if MH1=**0** and BMIc=**3** then MHMUs1=**5**;

if MH5=**1** and BMIc=**1** then MHMUs5=**1**;

else if MH5=**1** and BMIc=**2** then MHMUs5=**0**;

else if MH5=**1** and BMIc=**3** then MHMUs5=**2**;

else if MH5=**0** and BMIc=**1** then MHMUs5=**3**;

else if MH5=**0** and BMIc=**2** then MHMUs5=**4**;

else if MH5=**0** and BMIc=**3** then MHMUs5=**5**;

if MHMU4=0 and sex=0 then sexsubgr=0;

else if MHMU4=1 and sex=0 then sexsubgr=1; /*MHOOwomen*/

else if MHMU4=2 and sex=0 then sexsubgr=2; /*MUNWwomen*/

else if MHMU4=3 and sex=0 then sexsubgr=3; /*MUOOwomen*/

else if MHMU4=0 and sex=1 then sexsubgr=4; /*MHNWmen*/

else if MHMU4=1 and sex=1 then sexsubgr=5; /*MHOOmen*/

else if MHMU4=2 and sex=1 then sexsubgr=6; /*MUNWmen*/

else if MHMU4=3 and sex=1 then sexsubgr=7; /*MUOOmen*/

if MHMU4=0 and agec65=0 then agesubgr=0;

else if MHMU4=1 and agec65=0 then ageubgr=1; /*MHOOyoung*/

else if MHMU4=2 and agec65=0 then agesubgr =2; /*MUNWyoung*/

else if MHMU4=3 and agec65=0 then agesubgr =3; /*MUOOyoung*/

else if MHMU4=0 and agec65=1 then agesubgr =4; /*MHNWold*/

else if MHMU4=1 and agec65=1 then agesubgr =5; /*MHOOold*/

else if MHMU4=2 and agec65=1 then agesubgr =6; /*MUNWold*/

else if MHMU4=3 and agec65=1 then agesubgr =7; /*MUOOold*/

**run**;

/* outcome */

**%macro** oc(o);

data &o.;

set w.h_bhp_twhhh_v4;

if &o.=**.** then delete;

run;

**%mend** oc;

%***oc***(outcome1); /*5358(exclude underweight) 5719*/

/* Table1*/

**%macro** oc1(o);

/*table 1 */

proc means n mean std min median max data=&o.;

var age bmi WC_value sbp dbp Glu HbA1c TC TG HDL LDL;

run;

/*table 1 */

proc freq data=&o.;

table

agec gender BMIc smoker alc exercise menopausec CVDFHx marry edu incomec HRTuse;

run;

**%mend** oc1;

%***oc1***(outcome1);

/*table1 gr*/

**%macro** oc(o);

**%macro** g1(g1);

ods output ChiSq=chi;

proc freq data=&o.;

table (agec gender BMIc smoker alc exercise menopausec CVDFHx marry edu incomec)*&g1.

/chisq ;

run;

proc means n mean std median min max q1 q3 qrange data=&o.;

class &g1.;

var age bmi WC_value sbp dbp Glu HbA1c TC TG HDL LDL ;

var Cr eGFr;

run;

ods output out=a ModelANOVA=&o._f;

proc anova data=&o.;

class &g1.;

model

age bmi WC_value sbp dbp Glu HbA1c TC TG HDL LDL Cr eGFr=&g1.;

run;

ods output close;

proc print data=&o._f; run;

**%mend** g1;

%***g1***(MHMU4);

**%mend** oc;

%***oc***(outcome1);

/* Table 2 person-year */

**%macro** oc(o);

proc means n sum median max Q1 Q3 QRANGE data=&o.;

class MHMU4 ;

var &o._py ;

run;

**%mend** oc;

%***oc***(outcome1);

/*Table2 outcome frequency*/

**%macro** group(g);

**%macro** oc(o);

proc means n sum mean min median max Q1 Q3 QRANGE data=&o.;

class &g.;

var &o._py;

run;

proc freq data=&o.;

table &g.*&o.;

run;

**%mend** oc;

%***oc***(outcome1);

**%mend** group;

%***group***(MHMU4);

/* Table 2 Cox regression models*/

**%macro** model(o,m);

data t;

set &o;

/*if agec in ("0" "1");*/

/*if agec in ("2");*/

/*if agec14=1;*/

/*if agec in ("1");*/

/*if gender =1 ;*/

/*if smoker=0;*/

/*if exercise=0;*/

/*if &o._py<=1 then delete;*/

/*if menopause=1;*/

proc phreg;

class

agec(ref="0")

MHMU4(ref="0")

MHWC(ref="0")

/* MHMU_cal16(ref="0") MHMU_cal26(ref="0")*/

/* MHMUs1(ref="0")*/

MHMUs5(ref="0")

sexsubgr(ref="0")

agesubgr(ref="0")

; model &o._py*&o.(**0**) =

&m./RL;

run;

**%mend** model;

/*All models*/

/*outcome1 all 3 models*/

%***model***(outcome1, MHMU gender agec );

%***model***(outcome1, MHMU gender agec smoker alc exercise CVDFHx marry edu incomec);

%***model***(outcome1, MHMU gender agec smoker alc exercise CVDFHx marry edu incomec LDL );

%***model***(outcome4, MHMU1 gender agec smoker alc exercise marry edu incomec);

%***model***(outcome4, MHMU1 gender agec smoker alc exercise marry edu incomec);

/*subgroup analysis, change reference*/

%***model***(outcome1, sexsubgr agec smoker alc exercise CVDFHx marry edu incomec LDL );

%***model***(outcome1, agesubgr gender smoker alc exercise CVDFHx marry edu incomec LDL );

/* p for interaction */

%***model***(outcome1, MHMU1 gender agec smoker alc exercise CVDFHx marry edu incomec LDL);

%***model***(outcome1, MHMU1 gender agec smoker alc exercise CVDFHx marry edu incomec LDL gender*MHMU1);

%***model***(outcome1, MHMU1 gender agec smoker alc exercise CVDFHx marry edu incomec LDL smoker* MHMU1);

%***model***(outcome1, MHMU1 gender agec smoker alc exercise CVDFHx marry edu incomec LDL exercise*MHMU1);

%***model***(outcome1, MHMU1 gender agec65 smoker alc exercise CVDFHx marry edu incomec LDL);

%***model***(outcome1, MHMU1 gender agec65 smoker alc exercise CVDFHx marry edu incomec LDL agec65*MHMU1);

%***model***(outcome1, MHMU1 agec smoker alc exercise CVDFHx marry edu incomec LDL menopausec);

%***model***(outcome1, MHMU1 agec smoker alc exercise CVDFHx marry edu incomec LDL menopausec menopausec*MHMU1);

/*model for sensitivity of disease*/

%***model***(outcome1, MHMUs1 gender agec smoker alc exercise CVDFHx marry edu incomec LDL);

%***model***(outcome1, MHMUs2 gender agec smoker alc exercise CVDFHx marry edu incomec LDL);

%***model***(outcome1, MHMUs3 gender agec smoker alc exercise CVDFHx marry edu incomec LDL);

%***model***(outcome1, MHMUs4 gender agec smoker alc exercise CVDFHx marry edu incomec LDL);

%***model***(outcome1, MHMUs5 gender agec smoker alc exercise CVDFHx marry edu incomec LDL);

/* check PH assumption by time-dependent variables*/

**%macro** oc1(o);

data t;

set &o._1;

if MHMU4=**0** then MHMU4_0=**1**; else MHMU4_0=**0**;

if MHMU4=**1** then MHMU4_1=**1**; else MHMU4_1=**0**;

if MHMU4=**2** then MHMU4_2=**1**; else MHMU4_2=**0**;

if MHMU4=**3** then MHMU4_3=**1**; else MHMU4_3=**0**;

proc phreg ;

model &o._py*&o.(**0**) = MHMU4_1 MHMU4_2 MHMU4_3

MHMU4_1t MHMU4_2t MHMU4_3t ;

MHMU4_1t =MHMU4_1*log(&o._py);

MHMU4_2t =MHMU4_2*log(&o._py);

MHMU4_3t =MHMU4_3*log(&o._py);

proportionality_test: test MHMU4_1t, MHMU4_2t, MHMU4_3t ;

run;

**%mend** oc1;

%***oc1***(outcome1);

/* KM curve, output to STATA*/

**%macro** oc1(o);

data &o._1;

set &o.;

proc lifetest data=&o._1 plot=(s, lls) ; /* check PH assumption by log log plot */

time &o._py*&o.(**0**);

strata MHMU4;

run;

**%mend** oc1;

%***oc1***(outcome1);

/* output to STATA for KM curve*/

**%macro** oc1(o);

data t_&o.;

set &o.;

MHMU41=MHMU4;

if MHMU4="0" then MHMU41=**1**;

if MHMU4="1" then MHMU41=**0**;

keep MHMU41 &o. &o._py;

run;

PROC EXPORT DATA= WORK.t_&o.

OUTFILE= "C:\Users\H108044\Desktop\&o..csv"

DBMS=CSV REPLACE;

PUTNAMES=YES;

**%mend** oc1;

%***oc1***(outcome1);
