# Supplementary material for: Association between metabolically healthy obesity/overweight and cardiovascular disease risk: A representative cohort study in Taiwan
Source: PLoS One. 2021 Feb 1;16(2):e0246378. doi: 10.1371/journal.pone.0246378 (PMC7850496; doi:10.1371/journal.pone.0246378)
Supplement: Attachment — Submitted filename: response to revewsers.docx [file pone.0246378.s012.docx]

**PONE-D-20-26474
Association between metabolically healthy obesity/overweight and cardiovascular disease risk : A representative cohort study in Taiwan
PLOS ONE**

**Journal Requirements:**

1. Please ensure that your manuscript meets PLOS ONE's style requirements, including those for file naming. The PLOS ONE style templates can be found at

Response：Thanks for your advice. We revised our manuscript and references to meet the PLOS ONE's style requirements.

2. Thank you for stating the following financial disclosure:

"The funders had no role in study design, data collection and analysis, decision to publish, or preparation of the manuscript."

At this time, please address the following queries:

 Response：Thanks for your advice. We did not receive any funding for this study and stated “The authors received no specific funding for this work” in the cover letter.

3.  We note that you have indicated that data from this study are available upon request. PLOS only allows data to be available upon request if there are legal or ethical restrictions on sharing data publicly. For information on unacceptable data access restrictions, please see http://journals.plos.org/plosone/s/data-availability#loc-unacceptable-data-access-restrictions.

 Response：Thanks for your advice. The datasets generated and/or analysed during the current study are not publicly available due to the terms of consent to which the participants agreed but data are however available from the authors upon reasonable request and with permission of the Health Promotion Administration at the Ministry of Health and Welfare in Taiwan. We revised our cover letter and provided contact information for a data access committee, ethics committee, or other institutional body to which data requests may be sent.

**Reviewers' comments:**
1. Is the manuscript technically sound, and do the data support the conclusions?
The manuscript must describe a technically sound piece of scientific research with data that supports the conclusions. Experiments must have been conducted rigorously, with appropriate controls, replication, and sample sizes. The conclusions must be drawn appropriately based on the data presented.

Reviewer #1: Yes Reviewer #2: Yes

2. Has the statistical analysis been performed appropriately and rigorously?

Reviewer #1: Yes Reviewer #2: Yes

3. Have the authors made all data underlying the findings in their manuscript fully available?
The [PLOS Data policy](https://mxs.mailcloud.com.tw/mg-cgi/mg_redirect?dmn=mmh.org.tw&mid=5FA14D7A.0004C9CB&mtime=1604406650&url=http%3A%2F%2Fwww%2Eplosone%2Eorg%2Fstatic%2Fpolicies%2Eaction%23sharing) requires authors to make all data underlying the findings described in their manuscript fully available without restriction, with rare exception (please refer to the Data Availability Statement in the manuscript PDF file). The data should be provided as part of the manuscript or its supporting information, or deposited to a public repository. For example, in addition to summary statistics, the data points behind means, medians and variance measures should be available. If there are restrictions on publicly sharing data—e.g. participant privacy or use of data from a third party—those must be specified.

Reviewer #1: No Reviewer #2: Yes

4. Is the manuscript presented in an intelligible fashion and written in standard English?
PLOS ONE does not copyedit accepted manuscripts, so the language in submitted articles must be clear, correct, and unambiguous. Any typographical or grammatical errors should be corrected at revision, so please note any specific errors here.

Reviewer #1: Yes	Reviewer #2: Yes

5. Review Comments to the Author
Please use the space provided to explain your answers to the questions above. You may also include additional comments for the author, including concerns about dual publication, research ethics, or publication ethics. (Please upload your review as an attachment if it exceeds 20,000 characters)

**Reviewer #1:** This study examines risk of developing cardiovascular disease (CVD) in a cohort of people from Taiwan who were followed for ~14 years. The authors stratify subjects based on an index of metabolic health and whether they were normal weight or overweight/obese at baseline. It is reported that people that are metabolically healthy overweight/obese have a higher risk of developing CVD compared to people that were metabolically healthy normal weight at baseline. The magnitude of the increased CVD risk is in line with previous reports in other populations.
I have the following comments:
1) It is unclear why people with obesity and those that are overweight were combined. Was this due to low numbers of people with obesity in the study cohort?

Response：Thanks for your expert opinion. We combined people with obesity and those that are overweight due to low numbers of people with obesity in the study cohort. We revised our manuscript and added the baseline characteristics of metabolically healthy obesity and overweight separately in Table S5 and performed a sensitivity analysis according to the reviewer 2’s suggestion in Table S6.

Revised methods: Both metabolically healthy and unhealthy statuses were classified as normal weight and obesity/overweight. We combined the participants with obesity and overweight due to the small number of participants….

The baseline characteristics of 5 358 included individuals are listed in Table 1. The details information of obesity and overweight separately was in the S5 Table.

**Table S5. Baseline characteristics of metabolically healthy obesity and overweight participants**

|  | Metabolically healthy (n =491) | | Metabolically healthy  (n =1,720) | |  |
| --- | --- | --- | --- | --- | --- |
| Characteristics | Overweight  (n= 358) | Obesity  (n= 133) | Overweight  (n= 1,054) | Obesity  (n= 666) | *p* value |
|  | Mean (SD) | Mean (SD) | Mean (SD) | Mean (SD) |  |
| Age (years old) | 44.6 (13.1) | 40.1 (12.4) | 49.6 (14.1) | 47.5 (15.1) | **<0.001** |
| Body mass index (kg/m^2^ ) | 25.2 (0.8) | 29.6 (4.0) | 25.4 (0.9) | 29.7 (2.5) | **<0.001** |
| Waist circumference (cm) | 83.5 (6.7) | 92.5 (8.4) | 86.6 (6.9) | 95.7 (8.4) | **<0.001** |
| Systolic blood pressure (mmHg) | 109.4 (9.7) | 112.9 (8.9) | 124.1 (17.6) | 128.2 (17.6) | **<0.001** |
| Diastolic blood pressure (mmHg) | 72.5 (6.9) | 74.2 (6.4) | 80.7 (11.2) | 84.0 (11.3) | **<0.001** |
| Fasting plasma glucose (mg/dL) | 87.4 (6.4) | 88.1 (6.6) | 102.2 (35.1) | 107.4 (41.3) | **<0.001** |
| Hemoglobulin A1c (%) | 5.15 (0.52) | 5.22 (0.48) | 5.66 (1.24) | 5.86 (1.32) | **<0.001** |
| Total cholesterol (mg/dL) | 182.2 (26.2) | 184.8 (28.6) | 196.3 (40.6) | 200.8 (44.3) | **<0.001** |
| Triglycerides (mg/dL) | 94.1 (28.1) | 99.5 (26.9) | 178.0 (102.7) | 193.8 (109.8) | **<0.001** |
| High-density lipoprotein cholesterol (mg/dL) | 60.8 (11.5) | 60.8 (11.8) | 50.1 (15.7) | 48.8 (15.8) | **<0.001** |
| Low-density lipoprotein cholesterol (mg/dL) | 113.2 (20.0) | 114.9 (20.5) | 126 (28.4) | 129.3 (29.1) | **<0.001** |
|  | n (%) | n (%) | n (%) | n (%) |  |
| 20-39 (years old) | 134 (37.4) | 63 (47.0) | 273 (25.9) | 220 (33.1) | **<0.001** |
| 40-64 (years old) | 197 (54.9) | 68 (50.8) | 599 (56.9) | 345 (51.9) | **<0.001** |
| ≥ 65 (years old) | 28 (7.8) | 3 (2.3) | 181 (17.2) | 100 (15.0) | **<0.001** |
| Women | 194 (54.0) | 67 (50.0) | 637 (60.4) | 403 (60.5) | **<0.001** |
| Current smokers | 63 (17.6) | 31 (23.1) | 284 (27.0) | 196 (29.5) | **<0.001** |
| Alcohol used | 110 (30.6) | 35 (26.1) | 348 (33.1) | 218 (32.8) | **<0.001** |
| Regular exercise habit^a^ | 101 (28.2) | 30 (22.4) | 277 (26.3) | 144 (21.7) | **<0.001** |
| Menopaused status | 56 (15.6) | 21 (15.7) | 227 (21.6) | 149 (22.4) | **0.008** |
| Parental history of CVD | 87 (24.2) | 21 (15.8) | 291 (27.6) | 159 (23.9) | **<0.001** |
| Living with spouse | 269 (74.9) | 86 (64.2) | 804 (76.4) | 471 (70.8) | **<0.001** |
| Educational level ( ≥ 9 years of schooling) | 203 (56.6) | 75 (56.0) | 466 (44.3) | 295 (44.4) | **<0.001** |
| Average month income ≥ 40,000 NTD | 110 (30.6) | 28 (20.9) | 246 (23.4) | 138 (20.8) | **<0.001** |

Normal weight, 18.5 to 23.9 kg/m^2^; obesity/overweight, ≥24.0 kg/m^2^

^a^Regular exercise defined as more than 30 minutes a day, three times a week, lasting at least three months

SD: standard deviation

**Table S6. Subgroup analyses for fatal and nonfatal cardiovascular disease risk by metabolic statuses and body mass index categories by different reference groups.**

|  | Metabolically healthy | | Metabolically unhealthy | |
| --- | --- | --- | --- | --- |
| Variables | Normal weight | **Obesity/overweight** | Normal weight | Obesity/overweight |
| **Sex** |  |  |  |  |
| Women | 1 | **5.00 (1.74, 14.41)** | **5.77 (2.28, 14.59)** | **8.82 (3.54, 21.92)** |
| Men | **7.42 (2.81, 19.58)** | **8.96 (3.21, 25.07)** | **13.78 (5.51, 34.45)** | **15.15 (6.11, 37.54)** |
| **Age** |  |  |  |  |
| <65 years old | 1 | 2.06 (0.99, 4.28) | **4.47 (2.57, 7.78)** | **5.65 (3.31, 9.63)** |
| ≥65 years old | **16.04 (7.75, 33.22)** | **30.74 (14.42, 65.57)** | **21.63 (12.32, 37.97)** | **24.94 (14.30, 43.50)** |

2) MHO is used to mean both metabolically healthy obese and metabolically healthy overweight/obese. Perhaps the latter can be defined as MHOO to avoid confusion? Also obese is used is several places where people that are overweight are included.

Response：Thanks for your expert opinion. We revised our manuscript and changed the word MHO to MHOO to avoid confusion.

Revised sentence: Thus, we had four groups: MHNW (the reference group), MHOO (metabolically healthy obesity/overweight), …

3) In the Outcomes section study endpoints are CVD morbidity and mortality and cerebrovascular morbidity and mortality but I only see CVD morbidity data presented in the results section. If the Kaplan-Meier curves show mortality data this should be clearer as it reads as if these report time without CVD.

Response：Thanks for your expert opinion. We revised our manuscript and changed the CVD morbidity data presented in the results section to the CVD morbidity and mortality.

Revised sentence: We documented 439 incident cases of fatal and nonfatal CVD during a median… . The fatal and nonfatal CVD-free survival time … The adjusted fatal and nonfatal CVD HR ….

We revised the title of the figures and tables:

Fig 1. Kaplan–Meier survival curves of fatal and nonfatal cardiovascular disease outcome by metabolic status and body mass index category.

Table 2. Risk of fatal and nonfatal cardiovascular disease by metabolic status and body mass index categories.

Table 3. Subgroup analyses for fatal and nonfatal cardiovascular disease risk by metabolic statuses and body mass index categories.

4) The authors highlight differences in responses between women and men and in young vs older subjects. However, it is likely that the baseline risk was higher in men vs women and in older vs younger adults, which is lost when 1 is used as the reference value for all. It would be helpful to recalculate these risks all compared to the group with the lowest risk. So in Table 3 for the sex analysis all groups would likely be compared to women MHNW and for Age compared to the <65 year old MHNW group. Presenting results from this additional analysis would help the interpretation of the data.

Response：Thanks for your expert opinion. We revised our manuscript and changed the reference group in S7 Table.

Results: The fatal and nonfatal CVD risk in individuals with metabolically unhealthy profiles who were less than 65 years old was three to four folds, whereas in elderly individuals it was one to two folds. Smoking status did not change the point estimate significantly. In the subgroup analyses, in order to compare the fatal and nonfatal CVD risk between different sex and age groups, we changed the reference group to women with MHNW and, whom were less than 65 years old (S6 Table).

**Table S6. Subgroup analyses for fatal and nonfatal cardiovascular disease risk by metabolic statuses and body mass index categories by different reference groups.**

|  | Metabolically healthy | | Metabolically unhealthy | |
| --- | --- | --- | --- | --- |
| Variables | Normal weight | **Obesity/overweight** | Normal weight | Obesity/overweight |
| **Sex** |  |  |  |  |
| Women | 1 | **5.00 (1.74, 14.41)** | **5.77 (2.28, 14.59)** | **8.82 (3.54, 21.92)** |
| Men | **7.42 (2.81, 19.58)** | **8.96 (3.21, 25.07)** | **13.78 (5.51, 34.45)** | **15.15 (6.11, 37.54)** |
| **Age** |  |  |  |  |
| <65 years old | 1 | 2.06 (0.99, 4.28) | **4.47 (2.57, 7.78)** | **5.65 (3.31, 9.63)** |
| ≥65 years old | **16.04 (7.75, 33.22)** | **30.74 (14.42, 65.57)** | **21.63 (12.32, 37.97)** | **24.94 (14.30, 43.50)** |

5) In the discussion what support is there in humans that adipose tissue inflammation causes systemic inflammation and what “changes in adipose tissue composition” are there? Please be specific.

Response：Thanks for your expert opinion. We revised our discussion:

Genetic or functional variants of adipocytes cause less chronic inflammation and changes in adipose tissue composition from subcutaneous to visceral fat with variation in gastrointestinal microbiota as well as early life programing and less sedentary lifestyle could explain the mechanism of MHOO.[26, 27]

6) In the discussion the statement “the protective status of MHO is temporary” is too strong. It is true, and the authors mention later in the paragraph, that some people with MHO transition to metabolically unhealthy obesity over time but not all do.

Response：Thanks for your expert opinion. We revised our discussion: …However, the ~~protective~~ unharmful status of MHOO is temporary….

7) Why was LDL-cholesterol included as a covariate when this was included in the metabolically healthy criteria? Please include the rationale.

Response：Thanks for your expert opinion. In the metabolically healthy criteria, we exclude participants with existed hyperlipidemia, referred to a LDL-C level of ≥160 mg/dL in the first survey or long-term prescription of lipid-lowering agents. In the model 3, LDL-C level was served as a continuous variable, to adjust the different levels below 160 mg/dL. We added the description in the Table S4.

**Table S4. Definitions of the covariates in the study cohort**

| Covariates | Question in the questionnaire | Category |
| --- | --- | --- |
| Sex |  | Women/Men |
| Age | (Years old) | ≥20 to <40, ≥40 to <65, ≥65 |
| … | … | … |
| Low-density lipid cholesterol | (mg/dL) | Continuous variable |

8) Why was 2015 used as the study end point?

Response：Thanks for your expert opinion. This is the limitation of our cohort data, we revised the method: The clinical conditions were ascertained by reviewing the International Classification of Diseases, Ninth Revision-Clinical Modification (ICD-9-CM) codes obtained from the National Health Insurance Research Database from 2001 to 2015, the latest information released with permission from the Health Promotion Administration at the Ministry of Health and Welfare in Taiwan. (S3 Table).

9) Abstract – clarify that 8.6% is the prevalence of metabolically healthy overweight/obese in all subjects.

Response：Thanks for your expert opinion. We revised our abstract:

Among 5 358 subjects (mean [standard deviation] age, 44.5 [15.3] years; women, 48.2%), 1 479 were metabolically healthy with normal weight and 491 were metabolically healthy with obesity~~, with a prevalence of 8.6%~~. The prevalence of MHOO was 8.6% in the Taiwanese general population, which included individuals who were >20 years old, not pregnant, and did not have CVD (n = 5,719).

10) Waist circumference – In the abstract waist circumference is mentioned as being part of the inclusion criteria while in the methods section WC is mentioned as not being included. Which is correct?

Response：Thanks for your expert opinion. We revised our abstract:

Participants without diabetes, hypertension, and hyperlipidemia and who did not meet the metabolic syndrome without ~~and~~ waist circumference criteria were considered metabolically healthy.

11) Why was a cut-off of 160 mg/dl used for LDL-cholesterol? Less than 130 mg/dl is typically used as a normal concentration.

Response：Thanks for your expert opinion. According to the ATP III guidelines, LDL-c higher than 160 mg/dl was high and was more likely to be concerned as a disease status; LDL-c in130-159 mg/dl was considered as borderline high. We intended to exclude the hyperlipidemia disease status for the general population, thus we chose the cut-off of 160 mg/dl rather than 130 mg/dl.

12) Does “current smoking status” only relate to when the study started in 2002? Use of “current” make this confusing.

Response：Thanks for your expert opinion. The “current smoking status” only relate to when the study started in 2002, and we deleted the “current” in our manuscript.

**Table S4. Definitions of the covariates in the study cohort**

| Covariates | Question in the questionnaire | Category |
| --- | --- | --- |
| Sex |  | Women/Men |
| …. | … | … |
| ~~Current~~ Smoking status | Have you smoked before? 1. Never; 2. Yes, but only a few times;…. 1, 2 =~~current~~ smoker | ~~Current/Non-current smoker~~  Smoker/Non-smoker |

Table 3. Subgroup analyses for fatal and nonfatal cardiovascular disease risk by metabolic statuses and body mass index categories.

|  | Metabolically healthy | | Metabolically unhealthy | |
| --- | --- | --- | --- | --- |
| Variables | Normal weight | Obesity/overweight | Normal weight | Obesity/overweight |
| Smoker | 1 | 2.22 (0.80, 6.14) | 2.08 (1.03, 4.19) | 2.40 (1.22, 4.70) |
| Non-smoker | 1 | 1.79 (0.94, 3.43) | 2.80 (1.69, 4.63) | 3.54 (2.16, 5.79) |

**Reviewer #2**: This is a well-structured study with a rigorous analysis. I have only several minor comments:
(1) In line 100, the authors stated that "based on previous studies, ...", the references were lacking, please provide.

Response：Thanks for your expert opinion. We revised our method:

Based on previous studies[18-20], we selected the following potentially significant covariates for the analysis: basic demographics (sex and age).

Reference:

18.	Thomsen M, Nordestgaard BG. Myocardial infarction and ischemic heart disease in overweight and obesity with and without metabolic Syndrome. JAMA Internal Medicine. 2014;174(1):15-22. doi: 10.1001/jamainternmed.2013.10522.

19.	Ortega FB, Lee DC, Katzmarzyk PT, Ruiz JR, Sui X, Church TS, et al. The intriguing metabolically healthy but obese phenotype: Cardiovascular prognosis and role of fitness. European Heart Journal. 2013;34(5):389-97. doi: 10.1093/eurheartj/ehs174.

20.	Appleton SL, Seaborn CJ, Visvanathan R, Hill CL, Gill TK, Taylor AW, et al. Diabetes and cardiovascular disease outcomes in the metabolically healthy obese phenotype: A cohort study. Diabetes Care. 2013;36(8):2388-94. doi: 10.2337/dc12-1971.

(2) In Results sections, the description was too simplified, such as lines 161-164. I suggest to develop more in detail.

Response：Thanks for your expert opinion. We revised our results:

We documented 439 incident cases of fatal and nonfatal CVD in 67858.0 person-year during a median (interquartile range) follow-up time of 13.7 (13.6–13.8) years. The incident rate of fatal and nonfatal CVD was 6.47 per thousand person-year, including 259 carotid artery disease events, 209 stroke events, 4 fatal CAD, 9 fatal stroke, a person may had several events, but we calculated the first event as the person year. The fatal and nonfatal CVD-free survival time was significantly different in the four groups…

(3) Even though the authors indicated the limitation of not dividing obesity and overweight participants, I strongly comment to conduct a sensitivity analysis to evaluate the standard MHO groups without combining the overweight people.

Response：Thanks for your expert opinion. We conducted a sensitivity analysis to evaluate the standard MHO groups without combining the overweight people, only 3 events in the standard MHO group was noted.

Revised method: Several sensitivity analyses were performed to test the robustness of our results.… Finally, we separately estimated the hazard ratio of the metabolically healthy obesity and metabolically healthy overweight.

Revised result: Sensitivity analysis results are presented in Table S7-8 Tables..... However, the fatal and nonfatal CVD risk was not significantly in the metabolically healthy “obesity ” group (adjusted HR: 0.97, 95% CI: 0.30, 3.19), only 3 events developed in the group, but remained significantly higher in the metabolically healthy “overweight” group (adjusted HR: 2.03, 95% CI: 1.17, 3.53) (S8 Table).

**Table S8. Sensitivity analyses of the risk of fatal and nonfatal cardiovascular disease in metabolically healthy obesity and metabolically healthy overweight participants.**

|  | Metabolically healthy | |  | Metabolically unhealthy | |
| --- | --- | --- | --- | --- | --- |
| Variables | Normal weight | Overweight | Obesity | Normal weight | Obesity/overweight |
| Participants | 1,479 | 358 | 133 | 1,668 | 1,720 |
| Person-years | 19,786.7 | 4722.4 | 1799.6 | 20,715.6 | 20,841.6 |
| Events | 30 | 23 | 3 | 156 | 229 |
| Incidence rate  (per 1,000 person-years) | 1.52 | 4.87 | 1.67 | 7.53 | 10.97 |
| Model 1 | 1 | **2.00 (1.16, 3.48)** | 1.02 (0.31, 3.35) | **2.70 (1.82, 4.00)** | **3.50 (2.38, 5.14)** |
| Model 2 | 1 | **2.02 (1.16, 3.51)** | 0.99 (0.30, 3.26) | **2.64 (1.78, 3.92)** | **3.34 (2.27, 4.91)** |
| Model 3 | 1 | **2.03 (1.17, 3.53)** | 0.97 (0.30, 3.19) | **2.48 (1.65, 3.71)** | **3.09 (2.08, 4.58)** |
