# Supplementary material for: Association between metabolically healthy obesity/overweight and cardiovascular disease risk: A representative cohort study in Taiwan
Source: PLoS One. 2021 Feb 1;16(2):e0246378. doi: 10.1371/journal.pone.0246378 (PMC7850496; doi:10.1371/journal.pone.0246378)
Supplement: Attachment — Submitted filename: response to reviewers.docx [file pone.0246378.s013.docx]

6. Review Comments to the Author
Reviewer #1: The manuscript has been improved and the authors have adequately addressed my concerns with the exception of one minor concern listed below:

1) In response to the following comment “In the discussion the statement “the protective status of MHO is temporary” is too strong. It is true, and the authors mention later in the paragraph, that some people with MHO transition to metabolically unhealthy obesity over time but not all do.” The authors changed the sentence to read “However, the unharmful status of MHOO is temporary.” This still doesn’t reflect the fact that some MHOOs remain healthy over time and it isn’t “temporary”. Adding information that some people transition to a metabolically unhealthy state while others remain healthy would help clarify this statement.

Response：Thanks for your expert opinion. We revised our manuscript and changed the paragraph:

However, ~~the unharmful status of MHOO is temporary~~ some people transition to a metabolically unhealthy state while others remain healthy. Adipocyte tissue dysfunction and cytokine secretion lead to systematic inflammation, insulin resistance, and ultimately, transformation into a metabolically unhealthy status with diabetes and atherogenic dyslipidemia. This transformation results in the development of CVD. [31] Nearly 33%–52% of individuals with MHO undergo transition to metabolically unhealthy status in 6–20 years. [32]
